# Positive association between stress hyperglycemia ratio and ICU mortality in patients with pulmonary embolism: A retrospective study

**DOI:** 10.1371/journal.pone.0320644

**Published:** 2025-03-28

**Authors:** Jian Liao, Dingyu Lu, Maojuan Wang, Hanyang Yu

**Affiliations:** 1 Intensive Care Unit, Deyang People’s Hospital, Deyang, China; 2 Oncology Department, Deyang People’s Hospital, Deyang, China; 3 Emergency department, Deyang People’s Hospital, Deyang, China; University of Cape Town Faculty of Science, SOUTH AFRICA

## Abstract

**Background:**

Measurement of the Stress Hyperglycemia Ratio (SHR) aims to reduce the influence of prolonged chronic glycemic variables on stress hyperglycemia levels, which are associated with clinical outcomes. Nevertheless, the correlation between SHR and the risk of all-cause Intensive Care Unit (ICU) mortality in patients with pulmonary embolism(PE) remains unclear.

**Methods:**

Data for this retrospective study were o btained from the MIMIC IV2.2 database. The participants were divided into four groups based on the SHR quartiles. The primary outcome measured was 28-day ICU mortality. We employed Cox proportional hazards regression analysis and restricted cubic splines to evaluate the correlation between the SHR and clinical outcomes in patients with PE.

**Results:**

The study included 1185 patients, of which 53.3% were male. The rates of mortality observed in the ICU were 19.8%. By conducting multivariable Cox proportional hazards, it was determined that the SHR was independently associated with a heightened risk of 28-day ICU mortality (HR = 1.83 per 1-point increment, 95% CI = 1.07-3.13, p = 0.028).The analysis using restricted cubic splines showed that there was a consistent and gradually increasing risk of all-cause mortality as the SHR increased. This indicates that a higher SHR is associated with a higher risk of ICU mortality.

**Conclusions:**

Elevated SHR was strongly linked to a higher risk of clinical outcomes in patients with PE. As an effective measure of stress hyperglycemia, SHR demonstrated superior performance in predicting risks compared to solely evaluating glycemia or HbA1c upon admission.

## Introduction

PE is generally described as an obstruction in the pulmonary artery due to a clot, tumor, air or fat [1]. PE is the third most common cause of death amongst hospitalized patients [2]. Pulmonary embolism affects between 39 and 115 out of every 100,000 people each year, which greatly impacts medical care, hospital stay lengths, and death rates [3,4]. Overactivation of the sympathetic nervous system [5]and insulin resistance induced by stress [6,7] are frequently observed in patients admitted to the intensive care unit (ICU). This results in increased levels of hormones that elevate glucose (catecholamines, steroids, and glucagon), ultimately resulting in stress hyperglycemia [8]. Studies have shown that both acute and chronic elevation of blood glucose levels can lead to an increase in coagulation factors and a suppression of fibrinolysis. This, in turn, raises the risk of venous thromboembolism [9,10]. Elevated glucose levels have been linked to negative outcomes in various clinical conditions, including heart failure [11], acute myocardial infarction [12], ischemic [13] or hemorrhagic stroke [14].

Prior research often utilizes admission blood glucose (ABG) levels to define stress hyperglycemia. However, a heightened ABG reading may not consistently indicate an acute surge in glucose levels, especially among those with chronic hyperglycemia,such as diabetes,Thyroid Disorders and chronic hepatitis [15–18]. In order to accurately assess the prevalence of stress hyperglycemia, the SHR was introduced to minimize the influence of chronic glycemic factors on stress hyperglycemia levels [19]. The SHR assesses the level of stress-induced hyperglycemia relative to the seriousness of the illness, and has been proposed as a possible predictor of negative outcomes in critically ill patients [20–22]. However, there is a scarcity of research investigating the relationship between SHR values and the severity levels of PE. In order to address this gap, a retrospective cohort study was conducted to investigate the potential of the SHR in predicting all-cause mortality in patients with PE.

## Methods

### Study population

The researchers conducted a retrospective observational study using data from the publicly accessible Medical Information Mart for Intensive Care IV (MIMIC-IV) database, which can be found at https://mimic.mit.edu. Specifically, the study examined the medical records of patients in the ICU at Beth Israel Deaconess Medical Center from 2008 to 2019 [23]. In this study, data were analyzed retrospectively using an observational design. Ding yu Lu, as one of the authors, fulfilled the prerequisites to gain access to the database and undertook the task of data extraction. The patient cohort for this research comprised individuals with a confirmed diagnosis of acute pulmonary embolism, following the guidelines outlined in the International Classification of Diseases, 9th and 10th Revision. Ethical review and approval were waived for this study, due to reason: The use of the MIMIC-IV database was approved by the review committee of Massachusetts Institute of Technology and Beth Israel Deaconess Medical Center and patient’ data were anonymized prior to publication.This study followed the STROBE statement, and detailed results of filling out the STROBE checklist are provided in the attachment.

**Fig 1:**
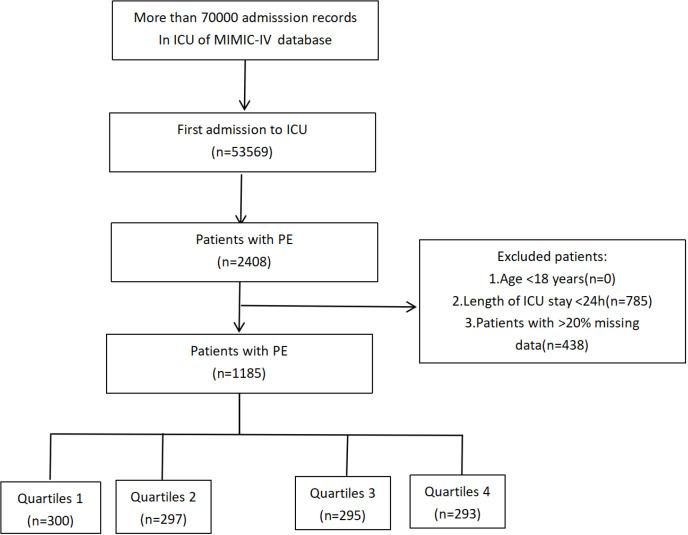
Flow of included patients through the trial.

The research excluded individuals below the age of 18 during their initial admission, individuals who experienced multiple admissions to the ICU due to PE (only data from the first admission were considered). We excluded patients who met the following criteria: less than 18 years (n = 0), length of ICU stay < 24h (n = 785), patients whose HbA1c or fasting blood glucose data within 24 hours of admission were unavailable. Finally, a total of 1185 patients formed the final study cohort and were divided into four groups based on the quartiles of the SHR observed on their first day in the ICU (Fig 1).

### Data collection

To conduct the data extraction, we utilized PostgresSQL (version 13.7.2, URL:https://www.postgresql.org/) software and pgAdmin 4 (version 7.5, URL:https://www.pgadmin.org/) tool by employing Structured Query Language (SQL). The extraction process prioritized four distinct categories of potential variables: demographic factors, vital signs, laboratory parameters, comorbidities and treatment during ICU stay. Vital signs and laboratory measurements from the initial 24 hours of ICU admission were included in the analysis. SHR was calculated as [(admission glucose (mg/dl))/(28.7 × HbA1c(%) - 46.7)] [19].

Exclusively acquired laboratory variables were obtained solely during the initial 24-hour period following patient admission. In instances where there were multiple outcomes, the average measurement was employed. To mitigate any potential bias, variables containing missing values surpassing 20% were eliminated, including C-reactive protein (CRP) and Interleukin-6 (IL-6) (S1 Table). To handle secondary variables (such as vital signs, complications and laboratory tests) with less than 20% missing data, we employed the multiple imputation (miss Forest R package) technique [24,25], five complete data sets each with 5 iterations were imputed with predictive mean matching using available covariates while excluding the outcome variables. Overall, the proportion of missing values among the vital signs and laboratory tests ranges from 12(1.01%) to 102(8.60%) out of 50,703 patients. This study extracted the primary variables and outcomes of each included patient, such as admission glucose, HbA1c(%) and ICU mortality. Therefore, no multiple imputation was performed on these complete variables.

### Outcomes

The main outcome of this study was ICU all-cause mortality at day 28. Secondary outcomes focused on hospital length of stay and ICU length of stay.

### Statistical analysis

Continuous variables were presented as the mean±SD or median and interquartile range (IQR). Categorical variables were expressed as numbers or percentages (%). ANOVA analysis, the Kruskal-Wallis test for continuous variables, or the chi-square test for categorical data, as applicable. The patients were categorized into four groups based on their admission SHR levels: Quartile 1 (SHR ≤ 0.72), Quartile 2 (0.72 < SHR ≤ 0.99), Quartile 3 (0.99 < SHR ≤ 1.36), and Quartile 4 (SHR > 1.36).

Kaplan-Meier survival analysis was used to assess the incidence rate of primary outcome events in different stratified groups based on the SHR. The log-rank test was employed to examine any observed disparities. Binary logistic regression analysis was conducted to evaluate factors influencing the risk of all-cause death.

Univariable Cox regression analysis was conducted to investigate the association between the SHR and the endpoints. Variables that showed clinical significance (p < 0.05) were included in the multivariable Cox proportional hazards model. When further evaluating the relationship between the SHR and ICU mortality, those with p <  0.05 in multivariate regression analysis were regarded as confounding factors to reduce the potential impact. (Model 1: unadjusted; Model 2: adjusted for Age, Gender and body mass index (BMI); Model 3: adjusted for Age, Gender, BMI, hemoglobin, fasting blood-glucose (FBG), HbA1c, lactate, activated partial thromboplastin time (APTT), blood urea nitrogen (BUN), hypertension, anticoagulant drugs, antiplatelet drugs, vasoactive drugs, hypotensive drugs, continuous renal replacement therapy (CRRT). HRs were counted and the findings were presented with 95% confidence intervals (CI). The lowest quartile of the SHR was used as the baseline group in all four models.

We also conducted an analysis to examine the non-linear relationship between the baseline SHR and ICU all-cause mortality. This analysis was done using a restricted cubic spline regression model with five knots.

In addition, Z test was used to compare the predictive value of SHR, blood glucose and HbA1c by comparing the area under curves (AUC) of the receiver operating characteristic curves (ROC).

Finally, subgroup analyses were conducted to investigate the consistency of the prognostic value of the SHR across different subgroups. These subgroups were categorized based on age (<65 versus ≥ 65 years), gender (female versus male) and the presence of specific medical histories including phlebothrombosis,

atrial fibrillation, hypertension, diabetes, whether to use vasoactive drugs and whether to use hypotensive drugs. Likelihood ratio tests were employed to evaluate the association between the SHR and the variables used for stratification. The data analyses were conducted using R software (version 4.2.2) and SPSS statistical software (Version 25.0). For all analyses, a 2-side P < 0.05 was considered statistically significant.

## Results

### Baseline characteristics

The study enrolled a total of 1185 critically ill patients with pulmonary embolism from the MIMIC-IV database, as depicted in Fig 1.

Table 1 presents the baseline characteristics of all patients. The median age of the study participants was 60 ±  14 years, with males comprising 53.3% of the sample. The ICU mortality rate was 19.8%. Patients in the quartile 4 group exhibited highest levels of fasting blood-glucose, lowest HbA1c, highest SHR and elevated levels of lactate and BUN. There was no statistical difference in comorbidities among the four groups(all p value > 0.05). However, there was a higher utilization of vasoactive drugs and hypotensive drugs among patients with a higher SHR. Moreover, patients with a highest SHR exhibited longer stays in the ICU (5 days, p = 0.049), and longer hospital stays (13 days, p = 0.042). The increase in SHR was also associated with increased ICU mortality (25.6% vs. 26.1% vs. 17.8% vs. 9.0%, p < 0.001).

**Table 1:** Baseline characteristics of participants stratified by SHR quartile levels.

Variable	Overall	Quartiles 1	Quartiles 2	Quartiles 3	Quartiles 4	P
		SHR ≤ 0.72	0.72 < SHR ≤ 0.99	0.99 < SHR ≤ 1.36	SHR > 1.36	
	n = 1185	n = 300	n = 297	n = 295	n = 293	
**Demographic**						
Age, years	60 ± 14	59 ± 15	59 ± 14	62 ± 13	61 ± 14	0.034
Male, n(%)	632 (53.3)	159 (53.0)	161 (54.2)	159 (53.9)	153 (52.2)	0.962
BMI	32 ± 11	32 ± 11	32 ± 12	31 ± 11	31 ± 11	0.767
**Vital signs**						
HR, beats/min	93 ± 17	93 ± 17	92 ± 17	93 ± 18	92 ± 18	0.792
SBP, mmHg	114 ± 16	114 ± 16	116 ± 16	115 ± 16	113 ± 16	0.310
DBP, mmHg	67 ± 12	67 ± 12	68 ± 12	66 ± 11	66 ± 12	0.060
RR, beats/min	20.7 ± 4.5	20.3 ± 4.4	20.8 ± 4.5	20.8 ± 4.7	21.0 ± 4.4	0.323
SPO2, %	96.36 ± 3.18	96.25 ± 3.54	96.57 ± 2.14	96.34 ± 3.18	96.29 ± 3.64	0.608
**Laboratory tests**						
WBC, K/uL	11 (8,16)	10 (7,15)	11 (8,16)	11 (8,16)	13 (9,18)	<0.001
Platelet, K/uL	201 (141, 278)	198 (142, 273)	206 (145, 280)	198 (131, 281)	196 (146, 274)	0.747
Hemoglobin, g/dL	10.40 (8.80, 12.10)	10.30 (8.60, 11.90)	10.50 (8.80, 12.10)	10.57 (8.85, 12.10)	10.57 (9.00, 12.60)	0.244
FBG, mg/dL	127 (104, 164)	103 (90, 118)	124 (102, 145)	137 (114, 177)	177 (138, 253)	<0.001
HbA1c, %	6.40 (5.20, 7.80)	7.90 (7.40, 8.83)	7.10 (5.60, 7.60)	5.50 (5.20, 7.20)	5.20 (4.20, 5.60)	<0.001
SHR	0.99 (0.72, 1.36)	0.57 (0.49, 0.64)	0.86 (0.79, 0.92)	1.18 (1.08, 1.27)	1.72 (1.51, 2.14)	<0.001
Lactate, mmol/L	2.20 (1.30, 2.40)	2.20 (1.20, 2.37)	2.10 (1.30, 2.37)	2.10 (1.30, 2.55)	2.37 (1.50, 2.90)	0.001
APTT, seconds	33 (28, 46)	36 (29, 48)	32 (27,45)	32 (28, 46)	33 (28, 48)	0.008
INR	1.41 (1.20, 1.64)	1.40 (1.22, 1.62)	1.42 (1.19, 1.64)	1.41 (1.20, 1.62)	1.43 (1.21, 1.65)	0.464
Triglyceride, mg/dL	242 (202, 284)	231 (194, 270)	238 (198, 279)	235 (201, 270)	237 (199, 275)	0.281
BUN, mg/dL	18 (13,29)	17 (11,28)	17 (12,28)	21 (14,34)	20 (14,30)	<0.001
Creatinine, mg/dL	0.90 (0.70, 1.40)	0.90 (0.70, 1.30)	0.90 (0.60, 1.30)	1.00 (0.70, 1.50)	1.00 (0.70, 1.50)	0.022
LDH, IU/L	456 (272, 581)	422 (250, 581)	456 (265, 581)	451 (282, 581)	500 (295, 581)	0.023
CK, IU/L	450 (145, 755)	455 (142, 769)	448 (173, 728)	452 (139, 766)	451 (141, 763)	0.433
CKMB, ng/mL	8.5 (5.0, 12.3)	8.4 (4.8, 11.9)	8.6 (5.1, 12.1)	8.5 (4.9, 12.2)	8.8 (5.2, 12.5)	0.482
TnT, ng/mL	0.31 (0.22, 0.41)	0.29 (0.20, 0.39)	0.32 (0.21, 0.44)	0.33 (0.23, 0.44)	0.30 (0.24, 0.37)	0.047
**Complication**						
Smoke, n(%)	193 (16.3)	48 (16.0)	53 (17.8)	49 (16.6)	43 (14.7)	0.770
Phlebothrombosis, n(%)	293 (24.7)	63 (21.0)	81 (27.3)	66 (22.4)	83 (28.3)	0.103
Atrial Fibrillation, n(%)	299 (25.2)	76 (25.3)	70 (23.6)	85 (28.8)	68 (23.2)	0.380
Hypertension, n(%)	425 (35.9)	115 (38.3)	115 (38.7)	94 (31.9)	101 (34.5)	0.246
Diabetes, n(%)	303 (25.6)	81 (27.0)	60 (20.2)	85 (28.8)	77 (26.3)	0.089
Heart failure, n(%)	343 (28.9)	72 (24.0)	85 (28.6)	96 (32.5)	90 (30.7)	0.117
AMI, n(%)	91 (7.7)	21 (7.0)	17 (5.7)	20 (6.8)	33 (11.3)	0.059
AKI, n(%)	457 (38.6)	119 (39.7)	108 (36.4)	115 (39.0)	115 (39.2)	0.840
CKD, n(%)	180 (15.2)	46 (15.3)	35 (11.8)	52 (17.6)	47 (16.0)	0.240
Liver cirrhosis, n(%)	66 (5.6)	16 (5.3)	13 (4.4)	23 (7.8)	14 (4.8)	0.265
Pneumonia, n(%)	441 (37.2)	108 (36.0)	103 (34.7)	109 (36.9)	121 (41.3)	0.376
Hyperlipemia, n(%)	1,072 (90.5)	266 (88.7)	264 (88.9)	270 (91.5)	272 (92.8)	0.414
Anticoagulant drugs, n(%)	1072 (90.5)	266 (88.7)	264 (88.9)	270 (91.5)	272 (92.8)	0.234
Antiplatelet drugs, n(%)	374 (31.6)	91 (30.3)	90 (30.3)	90 (30.5)	103 (35.2)	0.507
Vasoactive drugs, n(%)	537 (45.3)	116 (38.7)	124 (41.8)	146 (49.5)	151 (51.5)	0.004
Statins, n(%)	115 (9.7)	31 (10.3)	28 (9.4)	24 (8.1)	32 (10.9)	0.686
Hypotensive drugs, n(%)	806 (68.0)	186 (62.0)	193 (65.0)	214 (72.5)	213 (72.7)	0.007
Glucocorticoid, n(%)	409 (34.5)	103 (34.3)	95 (32.0)	96 (32.5)	115 (39.2)	0.235
Ventilation, n(%)	532 (44.9)	131 (43.7)	122 (41.1)	146 (49.5)	133 (45.4)	0.214
CRRT, n(%)	69 (5.8)	17 (5.7)	17 (5.7)	17 (5.8)	18 (6.1)	0.995
**Outcomes**						
ICU mortality, n(%)	235 (19.8)	27 (9.0)	53 (17.8)	77 (26.1)	78 (26.6)	<0.001
Hosp_day	12 (7,21)	11 (7,20)	11 (7,19)	13 (8,21)	13 (7,22)	0.042
ICU_day	4 (3,8)	4 (3,7)	4 (3,7)	5 (3,9)	5 (3,8)	0.005

BMI, body mass index; WBC, white blood cell; FBG, fasting blood-glucose; HR, heart rate; SBP, systolic blood pressure; DBP, diastolic blood pressure; RR, respiratory rate; HbA1c, hemoglobin A1c; SHR, stress hyperglycemia ratio; APTT, activated partial thromboplastin time; INR, international normalized ratio; BUN, blood urea nitrogen; LDH, lactate dehydrogenase; CK, creatine kinase; CKMB, creatinine kinase-myocardial band; TNT, troponin T; AMI, acute myocardial infarction; CKD, chronic kidney diseases; AKI, acute kidney injury; CRRT, continuous renal replacement therapy; Hosp_day, hospital length of stay; ICU_day, intensive care unit length of stay.

Table 2 presents the baseline characteristics comparing survivors to non-survivors. Compared with survivors, non-survivors tended to be older and have a higher proportion of heart failure, AMI, AKI, liver cirrhosis, pneumonia, hyperlipemia (all p < 0.05). Non-survivors were less likely to receive anticoagulant drugs, antiplatelet drugs and statins but more likely to receive ventilation and renal replacement therapy than survivors. For patients who died, there were higher levels of fasting blood glucose, activated partial thromboplastin time (APTT), international normalized ratio (INR), blood urea nitrogen (BUN), creatinine and lactate dehydrogenase (LDH) but lower levels of hemoglobin and HbA1c.

**Table 2:** Baseline characteristics of the Survivors and Non-survivors groups.

Variable	Overall n = 1185	Survivors n = 950	Non-survivors n = 235	P
**Demographic**				
Age, years	60 ± 14	60 ± 14	62 ± 13	0.026
Male,n(%)	632 (53.3)	512 (53.9)	120 (51.1)	0.436
BMI	32 ± 11	32 ± 11	31 ± 12	0.757
**Vital signs**				
HR, beats/min	93 ± 17	93 ± 17	92 ± 18	0.491
SBP, mmHg	114 ± 16	114 ± 16	116 ± 16	0.030
DBP, mmHg	67 ± 12	66 ± 12	67 ± 11	0.320
RR, beats/min	20.7 ± 4.5	20.8 ± 4.5	20.6 ± 4.5	0.731
SPO2, %	96.36 ± 3.18	96.36 ± 3.03	96.38 ± 3.74	0.922
**Laboratory tests**				
WBC, K/uL	11 (8,16)	11 (8,16)	12 (8,18)	0.140
Platelet, K/uL	201 (141, 278)	204 (144, 274)	190 (125, 285)	0.137
Hemoglobin, g/dL	10.40 (8.80, 12.10)	10.57 (8.90, 12.28)	10.00 (8.70, 11.80)	0.007
FBG, mg/dL	127 (104, 164)	125 (104, 161)	133 (109, 184)	0.014
HbA1c,%	6.40 (5.20, 7.80)	7.10 (5.30, 7.90)	5.80 (5.20, 6.95)	<0.001
SHR	0.99 (0.72, 1.36)	0.95 (0.68, 1.33)	1.16 (0.90, 1.53)	<0.001
Lactate, mg/dL	2.20 (1.30, 2.40)	2.30 (1.30, 2.37)	2.20 (1.50, 3.00)	0.011
APTT, seconds	33 (28, 46)	33 (28, 46)	37 (30, 55)	<0.001
INR	1.41 (1.20, 1.64)	1.30 (1.20, 1.62)	1.50 (1.30, 1.90)	<0.001
Triglyceride, mg/dL	242 (202, 284)	236 (197, 277)	239 (201, 279)	0.721
BUN, mg/dL	18 (13,29)	17 (12,27)	25 (15,42)	<0.001
Creatinine, mg/dL	0.90 (0.70, 1.40)	0.90 (0.70, 1.30)	1.10 (0.80, 1.70)	<0.001
LDH, IU/L	456 (272, 581)	435 (266, 581)	557 (308, 581)	<0.001
CK, IU/L	450 (145, 755)	453 (142, 766)	448 (150, 746)	0.713
CKMB, ng/mL	8.5 (5.0, 12.3)	8.2 (4.8, 11.7)	8.6 (5.0, 12.4)	0.217
TnT, ng/mL	0.29 (0.20, 0.39)	0.30 (0.22, 0.39)	0.32 (0.21, 0.44)	0.252
**Complication**				
Smoke, n(%)	293 (24.7)	232 (24.4)	61 (26.0)	0.733
Phlebothrombosis,n(%)	293 (24.7)	232 (24.4)	61 (26.0)	0.625
Atrial Fibrillation,n(%)	299 (25.2)	232 (24.4)	67 (28.5)	0.196
Hypertension,n(%)	425 (35.9)	336 (35.4)	89 (37.9)	0.474
Diabetes, n(%)	303 (25.6)	233 (24.5)	70 (29.8)	0.098
Heart failure, n(%)	343 (28.9)	258 (27.2)	85 (36.2)	0.006
AMI, n(%)	91 (7.7)	65 (6.8)	26 (11.1)	0.030
CKD, n(%)	180 (15.2)	143 (15.1)	37 (15.7)	0.791
AKI, n(%)	457 (38.6)	348 (36.6)	109 (46.4)	0.006
Liver cirrhosis, n(%)	66 (5.6)	46 (4.8)	20 (8.5)	0.028
Pneumonia, n(%)	441 (37.2)	340 (35.8)	101 (43.0)	0.041
Hyperlipemia, n(%)	347 (29.3)	265 (27.9)	82 (34.9)	0.035
**Treatment during hospitalization**				
Anticoagulant drugs,n(%)	1072 (90.5)	869 (91.5)	203 (86.4)	0.017
Antiplatelet drugs,n(%)	374 (31.6)	322 (33.9)	52 (22.1)	<0.001
Vasoactive drugs,n(%)	537 (45.3)	392 (41.3)	145 (61.7)	<0.001
Statins,n(%)	115 (9.7)	101 (10.6)	14 (6.0)	0.030
Hypotensive drugs,n(%)	806 (68.0)	640 (67.4)	166 (70.6)	0.336
Glucocorticoid,n(%)	409 (34.5)	317 (33.4)	92 (39.1)	0.095
Ventilation,n(%)	532 (44.9)	404 (42.5)	128 (54.5)	<0.001
CRRT,n(%)	69 (5.8)	45 (4.7)	24 (10.2)	0.001
**Outcomes**				
Hosp_day	12 (7,21)	13 (7,22)	9 (5,14)	<0.001
ICU_day	4 (3,8)	4 (3,8)	5 (3,9)	0.001

BMI, body mass index; WBC, white blood cell; FBG, fasting blood-glucose;HR, heart rate; SBP, systolic blood pressure; DBP, diastolic blood pressure; RR, respiratory rate; HbA1c, hemoglobin A1c; SHR, stress hyperglycemia ratio; APTT, activated partial thromboplastin time; INR, international normalized ratio; BUN, blood urea nitrogen; LDH, lactate dehydrogenase; CK, creatine kinase; CKMB, creatinine kinase-myocardial band; TNT, troponin T; AMI, acute myocardial infarction; CKD, chronic kidney diseases; AKI, acute kidney injury; CRRT, continuous renal replacement therapy; Hosp_day, hospital length of stay; ICU_day, intensive care unit length of stay.

### Primary outcomes

**Fig 2:**
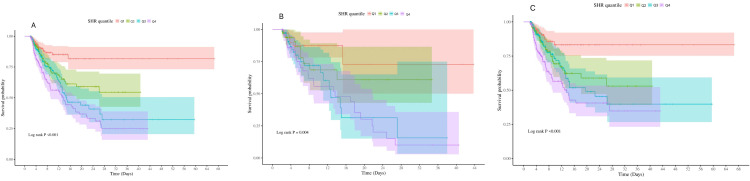
Kaplan–Meier survival analysis curves for all-cause 28-day ICU mortality. (A) Overall population; (B) patients with diabetes; (C) patients without diabetes; Q1:SHR ≤ 0.72; Q2:Quartiles 2:0.72 < SHR ≤ 0.99;Q3:Quartiles 3:0.99 < SHR ≤ 1.36;Q4:Quartiles 4:SHR > 1.36.

The ICU mortality at day 28 among groups was analyzed using Kaplan-Meier survival analysis curves, based on the SHR quartiles as presented in Fig 2. It was observed that patients with a higher SHR had a higher risk of ICU mortality (log-rank p < 0.0001).

Table 3 presents the results of the Cox regression conducted to assess the risk of all-cause death in patients with acute pulmonary embolism. The variables that showed significance in the univariate analysis (p < 0.05) and factors based on clinical experience were included as independent variables in the multivariate Cox regression analysis. Three models were employed: Model 1, which was unadjusted; Model 2, which was adjusted for Age, Gender and body mass index (BMI); and Model 3, which was adjusted for Age, Gender, BMI, hemoglobin, fasting blood-glucose (FBG), HbA1c, lactate, activated partial thromboplastin time (APTT), blood urea nitrogen (BUN), hypertension, anticoagulant drugs, antiplatelet drugs, vasoactive drugs, hypotensive drugs, continuous renal replacement therapy (CRRT). Cox proportional risk analysis was conducted to investigate the association between the SHR and 28-day ICU mortality. In the overall population, when the SHR was considered as a continuous variable, SHR was an independent risk factor for 28-day ICU mortality (HR = 1.83 per 1-point increment, 95% CI = 1.07–3.13, p = 0.028) after adjustment. When SHR was treated as a nominal variable, patients in the highest quartile group had a 4.25-fold higher risk of 28-day ICU mortality than those in the lowest quartile group. Similar associations were observed in the nondiabetic and diabetic populations (Table 4). These findings indicate a trend of increasing risk of mortality with higher SHR.

**Table 3:** Univariate and Multivariate Cox regression analysis for 28-day ICU mortality.

Variable	Univariate			Multivariate		
	HR	95%CI	P	HR	95%CI	P
Age	1.01	1.00, 1.02	0.077	1.00	0.99, 1.01	0.741
Male	0.92	0.72, 1.19	0.547	0.76	0.58, 1.01	0.060
BMI	0.99	0.98, 1.01	0.329	1.00	0.99, 1.01	0.705
HR	1.00	0.99, 1.00	0.413	1.00	0.99, 1.01	0.714
SBP	1.01	1.00, 1.01	0.077	1.01	1.00, 1.02	0.064
DBP	1.00	0.99, 1.01	0.482	1.00	0.98, 1.01	0.663
RR	1.00	0.97, 1.02	0.765	1.01	0.97, 1.04	0.671
SPO2	1.00	0.97, 1.04	0.883	1.00	0.96, 1.04	0.988
WBC	1.00	0.98, 1.01	0.745	0.99	0.98, 1.01	0.397
Platelet	1.00	1.00, 1.00	0.525	1.00	1.00, 1.00	0.190
Hemoglobin	0.90	0.85, 0.95	<0.001	0.91	0.85, 0.96	0.001
FBG	1.00	1.00, 1.00	0.194	0.99	0.99, 1.00	<0.001
HbA1c	0.85	0.78, 0.92	<0.001	1.24	1.05, 1.48	0.014
SHR	1.27	1.12, 1.44	<0.001	2.25	1.30, 3.89	0.004
SHR ≤ 0.72	reference					
0.72 < SHR ≤ 0.99	1.97	1.18, 3.29	0.010	2.94	1.63, 5.34	<0.001
0.99 < SHR ≤ 1.36	2.49	1.51, 4.11	<0.001	3.07	1.61, 5.87	<0.001
SHR ≥ 1.36	3.78	2.40, 5.96	<0.001	3.24	2.76, 6.17	<0.001
Lactate	1.07	1.02, 1.13	0.008	1.08	1.01, 1.15	0.029
APTT	1.01	1.00, 1.01	<0.001	1.01	1.00, 1.01	0.001
INR	1.09	1.02, 1.18	0.015	1.01	0.92, 1.11	0.773
Triglyceride	1.00	1.00, 1.00	0.543	1.00	1.00, 1.00	0.452
BUN	1.01	1.00, 1.01	<0.001	1.01	1.01, 1.02	<0.001
Creatinine	1.08	1.02, 1.15	0.007	0.94	0.86, 1.04	0.249
LDH	1.00	1.00, 1.00	0.230	1.00	1.00, 1.00	0.603
CK	1.00	1.00, 1.00	0.631	1.00	1.00, 1.00	0.405
CKMB	1.00	1.00, 1.01	0.329	1.00	1.00, 1.01	0.413
TnT	1.14	0.97, 1.35	0.110	1.14	0.96, 1.37	0.139
Smoke	1.01	0.72, 1.42	0.959	1.31	0.91, 1.88	0.145
Phlebothrombosis	1.01	0.75, 1.35	0.959	1.05	0.77, 1.45	0.749
Hypertension	1.16	0.89, 1.51	0.279	1.38	1.01, 1.90	0.044
Diabetes	1.20	0.91, 1.59	0.199	1.12	0.82, 1.52	0.478
Heart.failure	1.17	0.89, 1.52	0.254	1.00	0.73, 1.38	0.980
AMI	1.02	0.68, 1.54	0.916	1.12	0.72, 1.74	0.626
CKD	1.01	0.71, 1.44	0.955	0.87	0.57, 1.33	0.525
AKI	1.29	1.00, 1.67	0.051	1.20	0.90, 1.61	0.214
Liver cirrhosis	1.98	1.25, 3.13	0.004	1.54	0.93, 2.56	0.092
Pneumonia	1.22	0.94, 1.58	0.133	1.05	0.79, 1.39	0.725
Hyperlipemia	1.25	0.95, 1.63	0.105	1.36	1.00, 1.85	0.050
Anticoagulant.drugs	0.29	0.20, 0.43	<0.001	0.46	0.29, 0.71	<0.001
Antiplatelet.drugs	0.39	0.28, 0.53	<0.001	0.33	0.23, 0.47	<0.001
Vasoactive drugs	0.81	0.62, 1.07	0.137	0.66	0.48, 0.92	0.015
Statins	0.58	0.34, 1.00	0.049	0.81	0.46, 1.42	0.455
Hypotensive drugs	0.50	0.38, 0.67	<0.001	0.48	0.35, 0.66	<0.001
Glucocorticoid	1.10	0.85, 1.43	0.477	1.01	0.76, 1.36	0.925
Ventilation	1.10	0.85, 1.43	0.477	1.01	0.76, 1.36	0.925
CRRT	2.01	1.32, 3.06	0.001	2.13	1.32, 3.43	0.002

BMI, body mass index; WBC, white blood cell; FBG. fasting blood-glucose; HR, heart rate; SBP, systolic blood pressure; DBP, diastolic blood pressure; RR, respiratory rate; HbA1c, hemoglobin A1c; SHR, stress hyperglycemia ratio; APTT, activated partial thromboplastin time; INR, international normalized ratio; BUN, blood urea nitrogen; LDH, lactate dehydrogenase; CK, creatine kinase; CKMB, creatinine kinase-myocardial band; TNT, troponin T; AMI, acute myocardial infarction; CKD, chronic kidney diseases; AKI, acute kidney injury; CRRT, continuous renal replacement therapy; HR, hazard ratio; CI, confidence interval.

**Table 4:** Cox proportional hazard ratios (HR) for 28-day ICU mortality.

	Model 1		Model 2		Model 3	
Variable	HR (95%CI)	P	HR (95%CI)	P	HR (95%CI)	P
SHR as continuous	1.27 (1.12, 1.44)	<.001	1.46 (1.14, 2.15)	0.003	1.83 (1.07, 3.13)	0.028
Quartile 1	reference					
Quartile 2	1.97 (1.18, 3.29)	0.010	2.10 (1.24, 3.55)	0.006	3.12 (1.74, 5.59)	<.001
Quartile 3	2.47 (1.51, 4.03)	<.001	2.58 (1.52, 4.39)	<.001	3.57 (1.90, 6.69)	<.001
Quartile 4	3.96 (2.51, 6.26)	<.001	3.82 (3.08, 5.30)	<.001	4.25 (2.85, 6.24)	<.001
**Patients without diabetes**						
SHR as continuous	1.21 (1.04, 1.41)	0.016	1.43 (1.21, 2.03)	0.044	1.72 (0.50, 2.93)	0.031
Quartile 1	reference					
Quartile 2	2.14 (1.17, 3.92)	0.014	2.26(1.21, 4.21)	0.010	2.45(1.21, 4.47)	0.010
Quartile 3	2.36(1.30, 4.27)	0.005	2.56(1.36, 4.84)	0.004	2.84(1.35, 5.80)	0.004
Quartile 4	3.92(2.25, 6.82)	<.001	3.95(2.97, 5.64)	<.001	4.10(2.99, 5.86)	<.001
**Patients with diabetes**						
SHR as continuous	1.56(1.20, 2.03)	<.001	1.84(1.65, 2.18)	0.007	1.90(1.74, 2.45)	0.002
Quartile 1	reference					
Quartile 2	1.91(1.51, 2.92)	0.007	2.08(1.49, 3.94)	0.002	2.51(1.58, 3.99)	0.002
Quartile 3	2.69(1.13, 6.41)	0.025	2.44(0.94, 6.37)	0.068	3.00(1.11, 8.08)	0.030
Quartile 4	3.82(1.70, 8.60)	0.001	3.63(1.20, 9.03)	0.023	4.35(1.41, 10.67)	0.011

HR, hazard ratio; CI, confidence interval Model1: unadjusted.

Model 2: adjusted for Age, BMI, Gender.

Model3: Adjust: Age, Gender, BMI, HGB, FBG, HbA1c, Lac, APTT, BUN, Hypertension,Anticoagulant drugs, Antiplatelet drugs, Vasoactive drugs, Hypotensive drugs, CRRT.

Quartile 1: SHR ≤ 0.72; Quartile 2: 0.72 < SHR ≤ 0.99; Quartile 3: 0.99 < SHR ≤ 1.36; Quartile 4: SHR ≥ 1.36.

**Fig 3:**
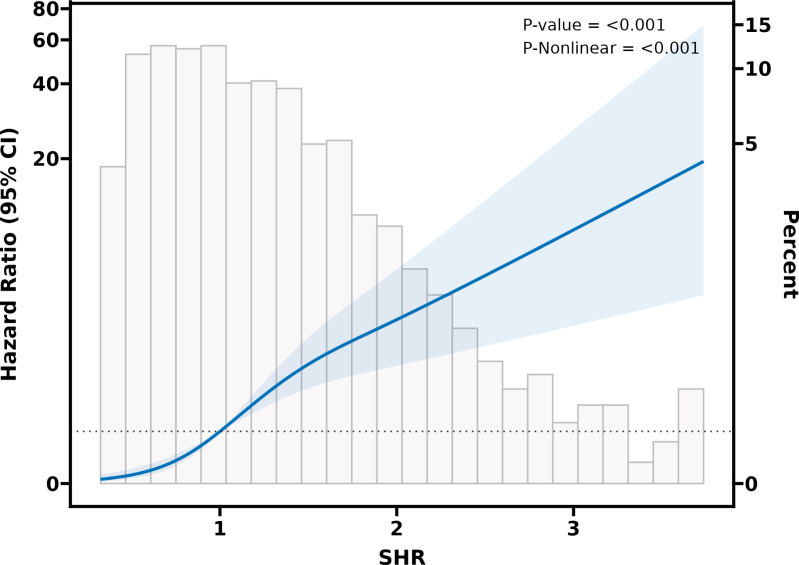
Multivariable-adjusted restricted cubic spline analyses of SHR for 28-day ICU mortality.

The study employed a restricted cubic splines regression model to investigate the relationship between SHR and the risk of 28-day ICU mortality. RCS curves showed a clear linear association between SHR and 28-day ICU mortality in patients with acute pulmonary embolism (Fig 3).

### Predictive accuracy of SHR for 28-day ICU mortality

**Fig 4:**
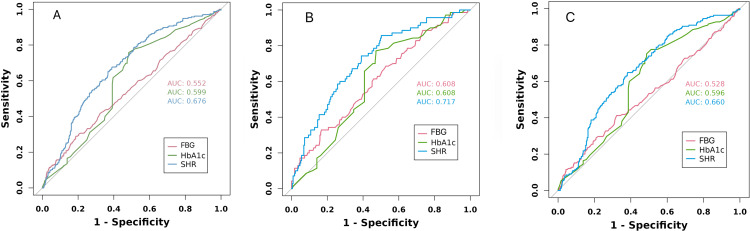
Receiver operating characteristic curves of SHR, FBG, and HbA1c in predicting 28-day ICU mortality. (A) Overall population; (B) patients without diabetes; (C) patients without diabetes; SHR, stress hyperglycemia ratio; FBG, fasting blood-glucose; HbA1c, hemoglobin A1c.

In overall population, the study compared SHR with FBG and HbA1c, showing that SHR (AUC = 0.676) had better predictive ability for composite events than blood glucose (AUC = 0.552) or HbA1c (AUC = 0.599). Similar results were observed in the non-diabetic and diabetic populations (Fig 4).

### Subgroup analysis

**Fig 5:**
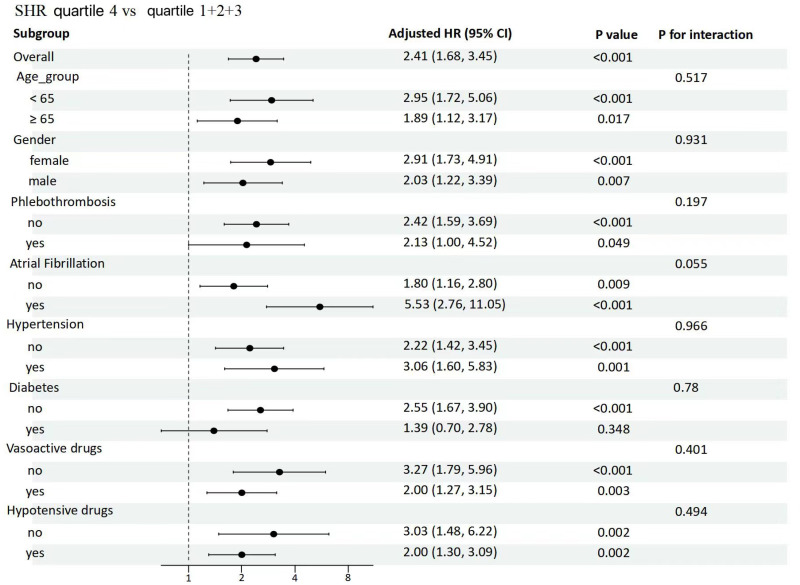
Forest plots for subgroup analyses of SHR with 28-day ICU mortality.

To investigate the relationship between SHR and 28-day ICU mortality, we estimated the relationship between SHR (≥1.36) and 28-day all-cause ICU mortality among subgroups, including age, gender, phlebothrombosis, atrial fibrillation, hypertension, diabetes, the use of vasoactive drugs and hypotensive drugs. The results showed that SHR (≥1.36) was associated with increased risk of 28-day all-cause mortality in the subgroups of age <  65 years [HR 2.95 (95% CI 1.72–5.06)], female [HR 2.91 (95% CI 1.73–4.91)], atrial fibrillation [HR 5.53 (95%CI 2.76–11.05)], hypertension [HR3.06 (95%CI 1.60–5.83)], non-phlebothrombosis [HR 2.42 (95%CI 1.59–3.69)], and non-diabetes [HR 2.55 (95% CI 1.67–3.90)], non-use of vasoactive drugs [HR 3.27 (95% CI 1.79–5.96)] and non-use of hypotensive drugs [HR 3.03 (95% CI 1.48–6.22)] (Fig 5).

## Discussion

To the best of our knowledge, this study represents the first investigation into the association between the SHR and all-cause mortality in patients with PE. The findings from this study suggest that a higher SHR is significantly correlated with increased ICU mortality rates among patients with PE. Notably, this association remained statistically significant even after adjusting for potential confounding risk factors. Consequently, these results indicate that the SHR holds promise as a valuable decision-making tool for clinicians and may serve as an independent prognostic factor in patients with PE.

Patients with pulmonary embolism often experience stress-induced hyperglycemia in the acute phase. These elevated levels can contribute to microvascular and macrovascular complications, as well as increase in coagulation factors, inhibition of fibrinolysis, platelet activation, and an inflammatory response, all of which elevate venous thromboembolism risk. [26–29]. However, a heightened ABG reading may not consistently indicate an acute surge in glucose levels, especially among those with chronic hyperglycemia [15–18]. Recent studies have confirmed the prognostic value of the SHR in various subpopulations with acute myocardial infarction (AMI) [30–33]. SHR proved more accurate in predicting hospital outcomes than ABG levels alone. Building upon these findings, our study focused on patients with PE and found that the risk of ICU mortality increased by 83% for these patients with each unit increase in the SHR index.

In our current investigation, an important linear correlation was identified between the SHR and 28-day ICU mortality. This indicates that the SHR has the potential to be a valuable tool in identifying individuals with a high risk of mortality.

Our results indicated a correlation between increased SHR levels and poorer functional status as well as unfavorable baseline characteristics. Furthermore, our results showed that SHR (≥1.36) was associated with increased risk of 28-day ICU mortality in the subgroups of age <  65 years, female, atrial fibrillation, hypertension, non-phlebothrombosis, and non-diabetes, non-use of vasoactive drugs and no-use of hypotensive drugs.

The link between high SHR and increased mortality can be explained in the following manner: (1) Excessive production of mitochondrial reactive oxygen species (ROS): Elevated levels of blood glucose lead to an excessive ROS production in the endothelial cells of both large and small blood vessels [34]; (2) The oxidative stress may cause dysfunction in endothelial cells, hindered vasodilation, and heightened vulnerability to cardiac incidents. Deficient fibrinolysis: hyperglycemia levels under stress commonly hinder the breakdown of blood clots. Reduced fibrinolysis and extended clot lysis duration are commonly observed characteristics of type 2 diabetes [9]. (3) Platelet activation is linked to hyperglycemia as a possible cause in vivo. It contributes to the nonenzymatic glycation of platelet glycoproteins, resulting in changes to the structure, conformation, and membrane lipid dynamics of platelets. This ultimately triggers their activation [35]. One of the primary advantages of SHR is its consideration of the patient’s baseline glycemic status. This is particularly important for critically ill patients, who frequently undergo acute stress responses that can result in transient hyperglycemia. Absolute glucose levels can be affected by a range of factors, including stress-induced hormonal changes, and may not accurately reflect the patient’s underlying metabolic state [26,27]. To summarize, the link between SHR and total mortality in patients may be due to the inflammatory reaction induced by fluctuations in blood glucose levels. Factors including the generation of ROS, compromised fibrinolysis, activation of platelets, dysfunction of the autonomic system, and complications unrelated to the cardiovascular system all play a role in the overall mortality.

Our study has several limitations. (1) As this study was retrospective in nature, we were unable to definitively establish causality. Despite the use of multivariate adjustment and subgroup analyses, there is still a possibility of residual confounding factors influencing the clinical outcomes. (2) SHR obtained from the initial measurements of blood glucose and HbA1c might not completely reflect the overall change in the body. It is important to consider this limitation when interpreting the results. (3) SHR is calculated using both a glucose measurement and an estimated average glucose derived from HbA1c levels. Consequently, the accuracy of SHR is dependent on the precision of both glucose and HbA1c measurements. Additionally, HbA1c levels are influenced by red blood cell lifespan, which can be significantly altered in critically ill patients, potentially affecting the reliability of SHR as a metric in this population. (4) Certain confounding factors such as inflammatory markers, metabolic syndrome parameters, nutritional state parameters, and Acute Physiology and Chronic Health Evaluation II (APACHE II) were not thoroughly taken into account, which could potentially influence the findings. (5) As the study data came from a single center (the MIMIC-IV database), our findings may not generalise to different healthcare settings, particularly those with different ICU resources or patient demographics. (6) With electronic health records data, there may be potential inaccuracies or inconsistencies in data entry, missing data, or variable measurements that may affect study validity. (7) Our study used multiple imputation, there could be limitations related to the accuracy of imputed data, especially if missingness was substantial. Therefore, further research is essential to comprehensively explore how this bias impacts clinical outcomes.

## Conclusions

Elevated SHR was strongly linked to a higher risk of longterm outcomes in patients with APE. As an effective measure of stress hyperglycemia, SHR demonstrated superior performance in predicting risks compared to solely evaluating glycemia or HbA1c upon admission.

## Supporting information

S1 Table Missing number for risk variables and outcome variables. (DOCX)

S1 File Data. (XLSX)

## References

[1] Rali P, Gandhi V, Malik K. Pulmonary embolism. Crit Care Nurs Q. 2016;39(2):131–8. doi: 10.1097/CNQ.0000000000000106 26919674

[2] Rali PM, Criner GJ. Submassive pulmonary embolism. Am J Respir Crit Care Med. 2018;198(5):588–98. doi: 10.1164/rccm.201711-2302CI 29672125

[3] Nguyen E, Caranfa JT, Lyman GH, Kuderer NM, Stirbis C, Wysocki M, et al. Clinical prediction rules for mortality in patients with pulmonary embolism and cancer to guide outpatient management: a meta-analysis. J Thromb Haemost. 2018;16(2):279–92. doi: 10.1111/jth.13921 29215781

[4] Konstantinides SV, Meyer G, Becattini C, Bueno H, Geersing G-J, Harjola V-P, et al. 2019 ESC Guidelines for the diagnosis and management of acute pulmonary embolism developed in collaboration with the European Respiratory Society (ERS). Eur Heart J. 2020;41(4):543–603. doi: 10.1093/eurheartj/ehz405 31504429

[5] Rizas KD, Nieminen T, Barthel P, Zürn CS, Kähönen M, Viik J, et al. Sympathetic activity-associated periodic repolarization dynamics predict mortality following myocardial infarction. J Clin Invest. 2014;124(4):1770–80. doi: 10.1172/JCI70085 24642467 PMC3973112

[6] Bartnik M, Malmberg K, Hamsten A, Efendic S, Norhammar A, Silveira A, et al. Abnormal glucose tolerance--a common risk factor in patients with acute myocardial infarction in comparison with population-based controls. J Intern Med. 2004;256(4):288–97. doi: 10.1111/j.1365-2796.2004.01371.x 15367171

[7] Wallander M, Bartnik M, Efendic S, Hamsten A, Malmberg K, Ohrvik J, et al. Beta cell dysfunction in patients with acute myocardial infarction but without previously known type 2 diabetes: a report from the GAMI study. Diabetologia. 2005;48(11):2229–35. doi: 10.1007/s00125-005-1931-z 16143862

[8] Shamoon H, Hendler R, Sherwin RS. Synergistic interactions among anti insulin hormones in the pathogenesis of stress hyperglycemia in humans. J Clin Endocrinol Metab. 1981;52(6):1235–41. 7014600 10.1210/jcem-52-6-1235

[9] Lemkes BA, Hermanides J, Devries JH. Hyperglycemia: a prothrombotic factor. J Thrombosis Haemost. 2010;8(8):1663–9. 10.1111/j.1538-7836.2010.03910.x 20492456

[10] Ageno W, Becattini C, Brighton T, Selby R, Kamphuisen PW. Cardiovascular risk factors and venous thromboembolism: a meta-analysis. Circulation. 2008;117(1):93–102. doi: 10.1161/CIRCULATIONAHA.107.709204 18086925

[11] Abdul-Quddus Mohammed A-Q, Luo Y, Wang K. Stress hyperglycemia ratio as a prognostic indicator for long-term adverse outcomes in heart failure with preserved ejection fraction. Cardiovascular Diabetology. 2024;23:275. 38350936 10.1186/s12933-024-02157-7 PMC10865536

[12] Gao S, Huang S, Lin X, Xu L, Yu M. Prognostic implications of stress hyperglycemia ratio in patients with myocardial infarction with nonobstructive coronary arteries. Ann Med. 2023;55(1):990–9. doi: 10.1080/07853890.2023.2186479 36896774 PMC10795641

[13] Roberts G, Sires J, Chen A, Thynne T, Sullivan C, Quinn S, et al. A comparison of the stress hyperglycemia ratio, glycemic gap, and glucose to assess the impact of stress-induced hyperglycemia on ischemic stroke outcome. J Diabetes. 2021;13(12):1034–42. doi: 10.1111/1753-0407.13223 34536055

[14] Wang Y, Cheng Y, Wu Q, Liu J, Liu M. High stress hyperglycemia ratio predicts hemorrhagic transformation after ischemic stroke. Journal of the Neurological Sciences. 2021;429:118706. doi: 10.1016/j.jns.2021.118706

[15] Kim EJ, Jeong MH, Kim JH, Ahn TH, Seung KB, Oh DJ, et al. Clinical impact of admission hyperglycemia on in-hospital mortality in acute myocardial infarction patients. Int J Cardiol. 2017;236:9–15. doi: 10.1016/j.ijcard.2017.01.095 28126258

[16] Paolisso P, Foà A, Bergamaschi L, Angeli F, Fabrizio M, Donati F, et al. Impact of admission hyperglycemia on short and long-term prognosis in acute myocardial infarction: MINOCA versus MIOCA. Cardiovasc Diabetol. 2021;20(1):192. doi: 10.1186/s12933-021-01384-6 34560876 PMC8464114

[17] Eom YS, Wilson JR, Bernet VJ. Links between Thyroid Disorders and Glucose Homeostasis. Diabetes Metab J. 2022;46(2):239–56. doi: 10.4093/dmj.2022.0013 35385635 PMC8987680

[18] Chen J, Wang F, Zhou Y, Jiang J, Ksimu S, Zhang X, et al. Chronic hepatitis C virus infection impairs insulin secretion by regulation of p38δ MAPK-dependent exocytosis in pancreatic β-cells. Clin Sci (Lond). 2020;134(5):529–42. doi: 10.1042/CS20190900 32100852

[19] Roberts GW, Quinn SJ, Valentine N, Alhawassi T, O’Dea H, Stranks SN, et al. Relative Hyperglycemia, a Marker of Critical Illness: Introducing the Stress Hyperglycemia Ratio. J Clin Endocrinol Metab. 2015;100(12):4490–7. doi: 10.1210/jc.2015-2660 26485219

[20] Zhou Y, Liu L, Huang H, Li N, He J, Yao H, et al. ’Stress hyperglycemia ratio and in-hospital prognosis in non-surgical patients with heart failure and type 2 diabetes. Cardiovasc Diabetol. 2022;21(1):290. doi: 10.1186/s12933-022-01728-w 36572923 PMC9791974

[21] Deng Y, Wu S, Liu J, Liu M, Wang L, Wan J, et al. The stress hyperglycemia ratio is associated with the development of cerebral edema and poor functional outcome in patients with acute cerebral infarction. Front Aging Neurosci. 2022;14:936862. doi: 10.3389/fnagi.2022.936862 36118702 PMC9474997

[22] Cui K, Fu R, Yang J, Xu H, Yin D, Song W, et al. Stress hyperglycemia ratio and long-term mortality after acute myocardial infarction in patients with and without diabetes: A prospective, nationwide, and multicentre registry. Diabetes Metab Res Rev. 2022;38(7):e3562. doi: 10.1002/dmrr.3562 35772392

[23] Johnson AEW, Bulgarelli L, Shen L, Gayles A, Shammout A, Horng S, et al. MIMIC-IV, a freely accessible electronic health record dataset. Sci Data. 2023;10(1):1. doi: 10.1038/s41597-022-01899-x 36596836 PMC9810617

[24] Blazek K, van Zwieten A, Saglimbene V, Teixeira-Pinto A. A practical guide to multiple imputation of missing data in nephrology. Kidney Int. 2021;99(1):68–74. doi: 10.1016/j.kint.2020.07.035 32822702

[25] Austin PC, White IR, Lee DS, van Buuren S. Missing Data in Clinical Research: A Tutorial on Multiple Imputation. Can J Cardiol. 2021;37(9):1322–31. doi: 10.1016/j.cjca.2020.11.010 33276049 PMC8499698

[26] Ishihara M. Acute hyperglycemia in patients with acute myocardial infarction. Circ J. 2012;76(3):563–71. doi: 10.1253/circj.cj-11-1376 22293452

[27] Das UN. Free radicals, cytokines and nitric oxide in cardiac failure and myocardial infarction. Mol Cell Biochem. 2000;215(1–2):145–52. doi: 10.1023/a:1026579422132 11204450

[28] Ujueta F, Weiss EN, Sedlis SP, Shah B. Glycemic Control in Coronary Revascularization. Curr Treat Options Cardiovasc Med. 2016;18(2):12. doi: 10.1007/s11936-015-0434-6 26820983

[29] Caturano A, Galiero R, Pafundi PC, Cesaro A, Vetrano E, Palmiero G, et al. Does a strict glycemic control during acute coronary syndrome play a cardioprotective effect? Pathophysiology and clinical evidence. Diabetes Res Clin Pract. 2021;178:108959. doi: 10.1016/j.diabres.2021.108959 34280467

[30] Lee TF, Burt MG, Heilbronn LK, Mangoni AA, Wong VW, McLean M, et al. Relative hyperglycemia is associated with complications following an acute myocardial infarction: a post-hoc analysis of HI-5 data. Cardiovasc Diabetol. 2017;16(1):157. doi: 10.1186/s12933-017-0642-3 29233143 PMC5725905

[31] Marenzi G, Cosentino N, Milazzo V, De Metrio M, Cecere M, Mosca S, et al. Prognostic Value of the Acute-to-Chronic Glycemic Ratio at Admission in Acute Myocardial Infarction: A Prospective Study. Diabetes Care. 2018;41(4):847–53. doi: 10.2337/dc17-1732 29382659

[32] Gao S, Liu Q, Ding X, Chen H, Zhao X, Li H. Predictive Value of the Acute-to-Chronic Glycemic Ratio for In-Hospital Outcomes in Patients With ST-Segment Elevation Myocardial Infarction Undergoing Percutaneous Coronary Intervention. Angiology. 2020;71(1):38–47. doi: 10.1177/0003319719875632 31554413 PMC6886151

[33] Xu W, Yang Y-M, Zhu J, Wu S, Wang J, Zhang H, et al. Predictive value of the stress hyperglycemia ratio in patients with acute ST-segment elevation myocardial infarction: insights from a multi-center observational study. Cardiovasc Diabetol. 2022;21(1):48. doi: 10.1186/s12933-022-01479-8 35351149 PMC8962934

[34] Giacco F, Brownlee M. Oxidative stress and diabetic complications. Circ Res. 2010;107(9):1058–70. doi: 10.1161/CIRCRESAHA.110.223545 21030723 PMC2996922

[35] Ferroni P, Basili S, Falco A, Davì G. Platelet activation in type 2 diabetes mellitus. J Thromb Haemost. 2004;2(8):1282–91. doi: 10.1111/j.1538-7836.2004.00836.x 15304032

