# Supplementary material for: Positive association between stress hyperglycemia ratio and ICU mortality in patients with pulmonary embolism: A retrospective study
Source: PLoS One. 2025 Mar 28;20(3):e0320644. doi: 10.1371/journal.pone.0320644 (PMC11952247; doi:10.1371/journal.pone.0320644)
Supplement: S1 Table — (DOCX) [file pone.0320644.s001.docx]

**Table S1.** missing number for risk variables and outcome variables

| **Variables** | Missing number (%) |
| --- | --- |
| **Demographic** |  |
| Age | 0 |
| Height | 86 (7.25%) |
| Weight | 95(8.01%) |
| Gender: male | 0 |
| BMI | 95(8.01%) |
| **Vital signs** |  |
| HR | 75(6.32%) |
| SBP | 34(2.86%) |
| DBP | 41(3.45%) |
| RR | 66(5.56%) |
| SPO2 | 12(1.01%) |
| **Complication** |  |
| Smoke | 0 |
| Phlebothrombosis | 0 |
| Atrial Fibrillation | 0 |
| Hypertension | 0 |
| Diabetes | 0 |
| Heart failure | 0 |
| AMI | 0 |
| AKI | 0 |
| CKD | 0 |
| Liver cirrhosis | 0 |
| Pneumonia | 0 |
| Hyperlipemia | 0 |
| **Treatment** |  |
| Anticoagulant drugs | 0 |
| Antiplatelet drugs | 0 |
| Vasoactive drugs | 0 |
| Statins | 0 |
| Hypotensive drugs | 0 |
| Glucocorticoid | 0 |
| Ventilation | 32(2.70%) |
| CRRT | 59(4.97%) |
| **Laboratory tests** |  |
| WBC | 0 |
| Platelet | 0 |
| Hemoglobin | 0 |
| FBG | 0 |
| HbA1c | 0 |
| SHR | 0 |
| Lactate | 38(3.20%) |
| APTT | 49(4.13%) |
| INR | 45(3.79%) |
| Triglyceride | 102(8.60%) |
| BUN | 20(1.68%) |
| Creatinine | 26(2.19%) |
| LDH | 58(4.89%) |
| CK | 37(3.12%) |
| CKMB | 37(3.12%) |
| TnT | 88(7.42%) |
| CRP (delete) | 472(39.83%) |
| IL-6 (delete) | 745(62.86%) |
| **Outcomes** |  |
| ICU mortality | 0 |
| Hospital_day | 0 |
| ICU_day | 0 |

Abbreviation:BMI: Body Mass Index; WBC: White Blood Cell;FBG: fasting blood-glucose;HR: Heart Rate; SBP:SystolicBlood Pressure; DBP;Diastolic Blood

Pressure; RR:Respiratory Rate;HbA1c: hemoglobin A1c; SHR:Stress Hyperglycemia Ratio; APTT:Activated Partial Thromboplastin Time; INR:International

Normalized Ratio; BUN:Blood Urea Nitrogen; LDH:Lactate Dehydrogenase; CK:Creatine Kinase; CKMB:Creatine Kinase-MB;TNT:Troponin T; AMI:Acute

Myocardial Infarction; CKD:Chronic Kidney Diseases; AKI:Acute Kidney Injury; CRRT:Continuous Renal Replacement Therapy; Hosp_day:Hospital Length of Stay;

ICU_Los,Day: Intensive Care Unit Length of Stay.
